# (*E*)-4-(2-Hy­droxy-3-meth­oxy­benzyl­idene­amino)-6-methyl-3-sulfanyl­idene-3,4-dihydro-1,2,4-triazin-5(2*H*)-one

**DOI:** 10.1107/S1600536812036756

**Published:** 2012-08-31

**Authors:** Bahareh Shirinkam, Masoumeh Tabatabaee, Mitra Gassemzadeh, Bernhard Neumuller

**Affiliations:** a Department of Chemistry, Yazd Branch, Islamic Azad University, Yazd, Iran; b Chemistry and Chemical Engineering Research Center of Iran, Tehran, Iran; c Department of Chemistry, Marburg University, Marburg, Germany

## Abstract

In the title mol­ecule, C_12_H_12_N_4_O_3_S, there is an intra­molecular O—H⋯N hydrogen bond. The dihedral angle between the benzene and triazine rings is 65.9 (3)°. In the crystal, N—H⋯S and O—H⋯N hydrogen bonds link the mol­ecules into chains along [010]. In addition, there are weak π–π stacking inter­actions between symmetry-related triazine rings with a centroid–centroid distance of 3.560 (3)°.

## Related literature

For the biological activity of azomethine compounds, see: Todeschini *et al.* (1998 ▶); Demirbas (2004 ▶); Rando *et al.* (2002 ▶). For general applications of Schiff base compounds, see: Galic *et al.* (2001 ▶); Wyrzykiewicz & Prukah (1998 ▶); Dubey *et al.* (1991 ▶). For the crystal structures of related Schiff base compounds, see: Tabatabaee *et al.* (2006 ▶, 2007 ▶, 2008 ▶, 2009 ▶). For the synthesis of the starting material, see: Dornow *et al.* (1964 ▶).
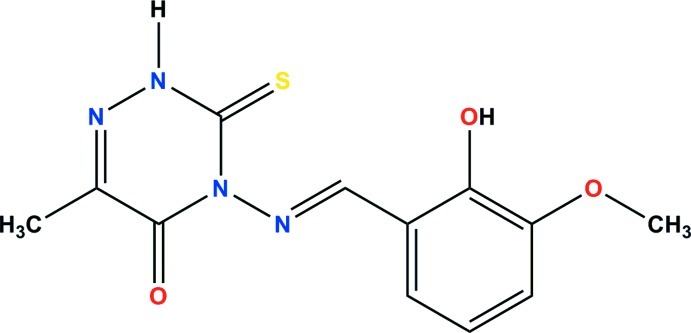


## Experimental

### 

#### Crystal data

C_12_H_12_N_4_O_3_S
*M* *_r_* = 292.32
Monoclinic, 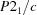

*a* = 13.679 (3) Å
*b* = 6.799 (1) Å
*c* = 13.797 (3) Å
β = 97.37 (2)°
*V* = 1272.6 (4) Å^3^
*Z* = 4
Mo *K*α radiation
μ = 0.27 mm^−1^
*T* = 100 K
0.17 × 0.16 × 0.05 mm

#### Data collection

Stoe IPDS II diffractometer
Absorption correction: numerical (*X-SHAPE* and *X-RED32*; Stoe & Cie, 2008 ▶) *T*_min_ = 0.19, *T*_max_ = 1.0
6252 measured reflections
2466 independent reflections
781 reflections with *I* > 2σ(*I*)
*R*_int_ = 0.168

#### Refinement

*R*[*F* ^2^ > 2σ(*F* ^2^)] = 0.063
*wR*(*F* ^2^) = 0.148
*S* = 0.69
2466 reflections
184 parameters
H-atom parameters constrained
Δρ_max_ = 0.25 e Å^−3^
Δρ_min_ = −0.25 e Å^−3^

### 

Data collection: *X-AREA* (Stoe & Cie, 2008 ▶); cell refinement: *X-AREA*; data reduction: *X-RED32* (Stoe & Cie, 2008 ▶); program(s) used to solve structure: *SIR92* (Altomare *et al.*, 1994 ▶); program(s) used to refine structure: *SHELXL97* (Sheldrick, 2008 ▶); molecular graphics: *SHELXTL* (Sheldrick, 2008 ▶) and *PLATON* (Spek, 2009 ▶); software used to prepare material for publication: *WinGX* (Farrugia, 1999 ▶).

## Supplementary Material

Crystal structure: contains datablock(s) I, global. DOI: 10.1107/S1600536812036756/lh5515sup1.cif

Structure factors: contains datablock(s) I. DOI: 10.1107/S1600536812036756/lh5515Isup2.hkl

Supplementary material file. DOI: 10.1107/S1600536812036756/lh5515Isup3.cml

Additional supplementary materials:  crystallographic information; 3D view; checkCIF report

## Figures and Tables

**Table 1:** Hydrogen-bond geometry (Å, °)

*D*—H⋯*A*	*D*—H	H⋯*A*	*D*⋯*A*	*D*—H⋯*A*
N4—H1⋯S1^i^	0.88	2.45	3.287 (5)	160
O2—H2⋯N2	0.89	1.87	2.662 (7)	146
O2—H2⋯N3^ii^	0.89	2.57	3.135 (7)	122

Symmetry codes: (i) 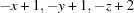; (ii) 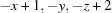.

**Fig. 1.**
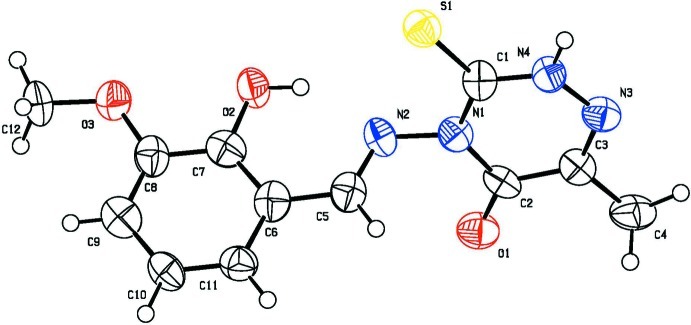
The molecular structure of the title compound with displacement ellipsoids drawn at the 50% probability level.

**Fig. 2.**
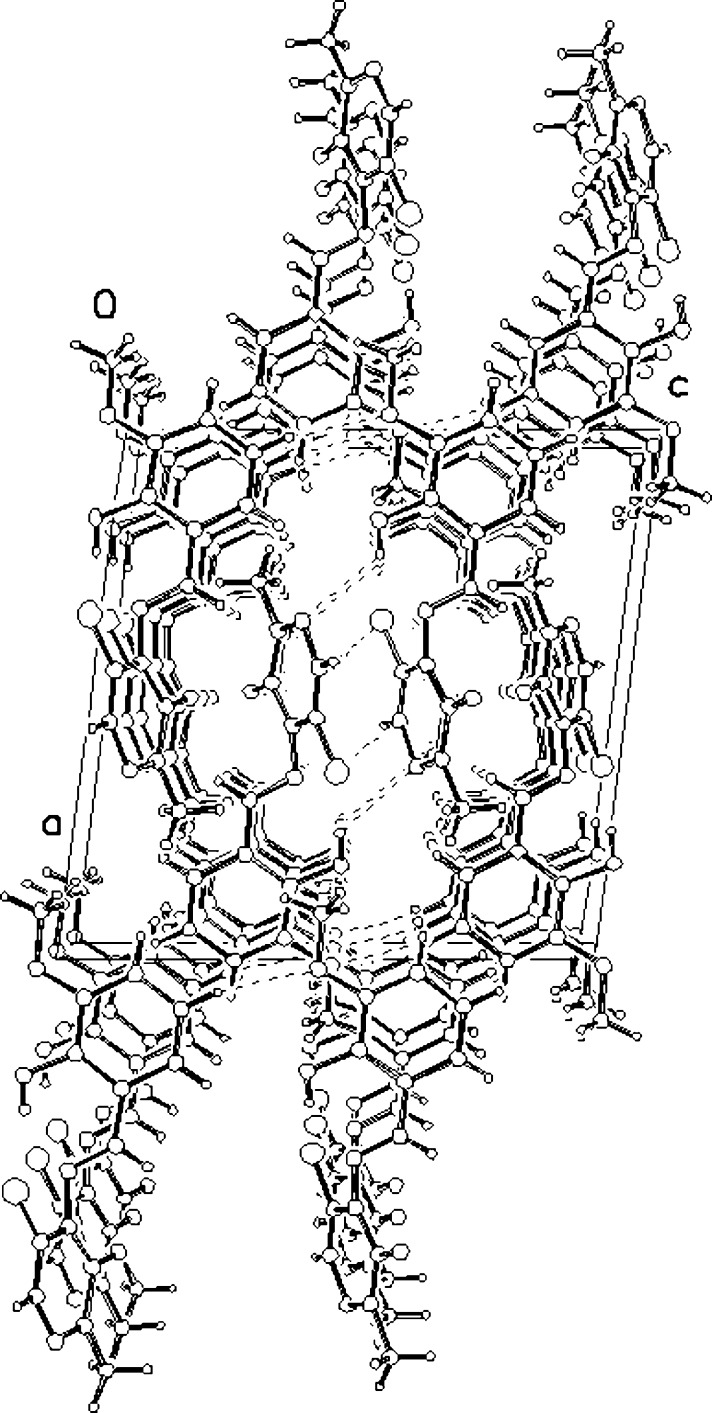
Part of the crystal structure with hydrogen bonds drawn as dashed lines.

**Fig. 3.**
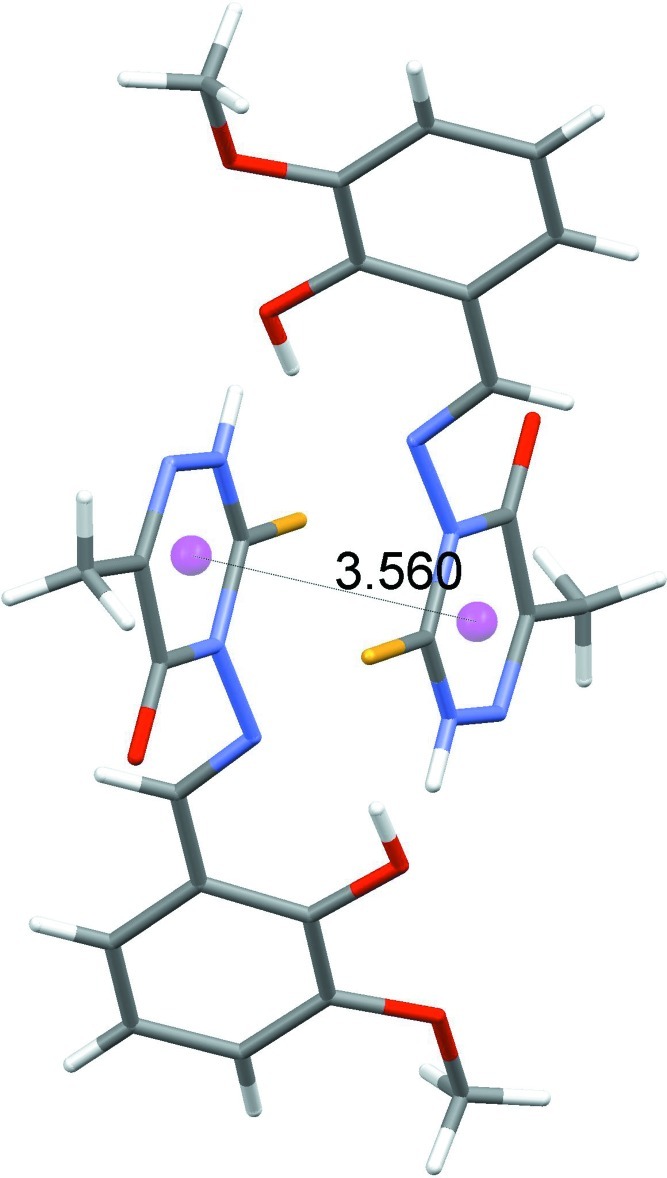
A view of a π–π stacking interaction.

## supplementary crystallographic information

### Comment

Azomethine compounds have been extensively studied for various reasons, one of which is their biological activity (Todeschini *et al.*, 1998; Demirbas, 2004; Rando *et al.*, 2002). Schiff bases, containing different donor atoms, also find use in analytical applications and metal coordination (Galic *et al.*, 2001; Wyrzykiewicz & Prukah, 1998; Dubey *et al.*, 1991). In a sequence of studies, we have investigated the synthesis and crystal structure of several Schiff bases derived 4-amino-5-methyl-2*H*-1,2,4-triazole-3(4*H*)-thione (AMTT) and 4-amino-6-methyl-3-thio-3,4-dihydro-1,2,4-triazin-5(2*H*)-one (AMTTO) compounds (Tabatabaee *et al.* 2006;2007;2008;2009) with various aldehydes. Herein, we report the crystal structure of the title compound.

The molecular structure of the title compound is shown in Fig. 1. The bond distances and angles agree with related compounds (Tabatabaee *et al.*, 2006; 2007; 2008; 2009). In the crystal, N—H···S and O—H···N hydrogen bonds link molecules into chains along [010] (Fig. 2). In addition, there are weak π–π stacking interactions between triazine rings Cg···Cg(1-*x*,-y,2-*z*) = 3.560 (3)Å, where Cg is the centroid defined by N1/C1/N4/N3/C3/C2.

### Experimental

4-Amino-6-methyl-3-thioxo-3,4-dihydro-1,2,4-triazin-5(2*H*)-one (AMTTO) was prepared according to the literature procedure (Dornow *et al.*, 1964). A solution of (AMTTO) (0.632 g, 4 mmol) in ethanol (10 ml) was treated with 2-hydroxy-3-methoxybenzaldehyde (0.608 g, 4 mmol) and the resulting mixture was acidified with 3 drops of hydrochloric acid (37.5%). The reaction mixture was refluxed. The progress of the reaction was monitored by TLC. After completion of the reaction (8 hrs), the pale yellow precipitate was filtered off and the clear solution was kept at 277K to give yellow plates crystals of the title compound (yield 79%).

### Refinement

H atoms were placed in calculated positions with C—H = 0.95 - 0.98 Å, O—H = 0.89Å and N—H = 0.88Å. They were included in the refinement in a riding-motion approximation with a refined common displacement parameter of U_iso_(H) = 0.062 (6)Å^2^. The diffraction from the crystal was very weak and this may affect the precision of the structure.

### Crystal data

**Table:**

C_12_H_12_N_4_O_3_S	*F*(000) = 608
*M**_r_* = 292.32	*D*_x_ = 1.526 Mg m^−^^3^
Monoclinic, *P*2_1_/*c*	Mo *K*α radiation, λ = 0.71073 Å
Hall symbol: -P 2ybc	Cell parameters from 2500 reflections
*a* = 13.679 (3) Å	θ = 1.5–25.9°
*b* = 6.799 (1) Å	µ = 0.27 mm^−^^1^
*c* = 13.797 (3) Å	*T* = 100 K
β = 97.37 (2)°	Plate, yellow
*V* = 1272.6 (4) Å^3^	0.17 × 0.16 × 0.05 mm
*Z* = 4	

### Data collection

**Table:**

Stoe IPDS II diffractometer	2466 independent reflections
Radiation source: fine-focus sealed tube	781 reflections with *I* > 2σ(*I*)
Graphite monochromator	*R*_int_ = 0.168
ω scans	θ_max_ = 25.9°, θ_min_ = 1.5°
Absorption correction: numerical (*X-SHAPE* and *X-RED32*; Stoe & Cie, 2008)	*h* = −16→16
*T*_min_ = 0.19, *T*_max_ = 1.0	*k* = −8→7
6252 measured reflections	*l* = −16→16

### Refinement

**Table:**

Refinement on *F*^2^	Primary atom site location: structure-invariant direct methods
Least-squares matrix: full	Secondary atom site location: difference Fourier map
*R*[*F*^2^ > 2σ(*F*^2^)] = 0.063	Hydrogen site location: inferred from neighbouring sites
*wR*(*F*^2^) = 0.148	H-atom parameters constrained
*S* = 0.69	*w* = 1/[σ^2^(*F*_o_^2^) + (0.0454*P*)^2^] where *P* = (*F*_o_^2^ + 2*F*_c_^2^)/3
2466 reflections	(Δ/σ)_max_ = 0.002
184 parameters	Δρ_max_ = 0.25 e Å^−^^3^
0 restraints	Δρ_min_ = −0.25 e Å^−^^3^

### Special details

**Table:**

Geometry. All s.u.'s (except the s.u. in the dihedral angle between two l.s. planes) are estimated using the full covariance matrix. The cell s.u.'s are taken into account individually in the estimation of s.u.'s in distances, angles and torsion angles; correlations between s.u.'s in cell parameters are only used when they are defined by crystal symmetry. An approximate (isotropic) treatment of cell s.u.'s is used for estimating s.u.'s involving l.s. planes.
Refinement. Refinement of *F*^2^ against ALL reflections. The weighted *R*-factor *wR* and goodness of fit *S* are based on *F*^2^, conventional *R*-factors *R* are based on *F*, with *F* set to zero for negative *F*^2^. The threshold expression of *F*^2^ > 2σ(*F*^2^) is used only for calculating *R*-factors(gt) *etc*. and is not relevant to the choice of reflections for refinement. *R*-factors based on *F*^2^ are statistically about twice as large as those based on *F*, and *R*- factors based on ALL data will be even larger.

### Fractional atomic coordinates and isotropic or equivalent isotropic displacement parameters (Å^2^)

**Table:**

	*x*	*y*	*z*	*U*_iso_*/*U*_eq_	
S1	0.63854 (13)	0.3469 (2)	0.97729 (12)	0.0493 (5)	
O1	0.4940 (3)	−0.2444 (6)	0.8000 (3)	0.0543 (12)	
O2	0.8173 (3)	−0.1839 (6)	1.0066 (3)	0.0502 (11)	
H2	0.7563	−0.1360	0.9959	0.062 (6)*	
O3	1.0050 (3)	−0.2641 (5)	1.0104 (3)	0.0533 (11)	
N1	0.5557 (4)	0.0250 (6)	0.8869 (3)	0.0424 (12)	
N2	0.6523 (4)	−0.0561 (6)	0.9024 (4)	0.0448 (13)	
N3	0.3669 (4)	0.1720 (7)	0.8798 (3)	0.0466 (13)	
N4	0.4513 (4)	0.2673 (6)	0.9210 (3)	0.0425 (13)	
H1	0.4422	0.3832	0.9467	0.062 (6)*	
C1	0.5420 (5)	0.2093 (8)	0.9268 (4)	0.0450 (16)	
C2	0.4779 (5)	−0.0841 (8)	0.8374 (4)	0.0417 (15)	
C3	0.3819 (4)	0.0048 (8)	0.8378 (4)	0.0451 (16)	
C4	0.2920 (5)	−0.1023 (8)	0.7908 (4)	0.0570 (19)	
H41	0.2328	−0.0300	0.8028	0.062 (6)*	
H42	0.2905	−0.2346	0.8188	0.062 (6)*	
H43	0.2943	−0.1121	0.7203	0.062 (6)*	
C5	0.6952 (5)	−0.0721 (8)	0.8260 (5)	0.0473 (17)	
H51	0.6594	−0.0394	0.7645	0.062 (6)*	
C6	0.7947 (5)	−0.1371 (9)	0.8290 (5)	0.0478 (16)	
C7	0.8550 (5)	−0.1852 (9)	0.9182 (5)	0.0504 (17)	
C8	0.9545 (5)	−0.2297 (8)	0.9195 (5)	0.0491 (17)	
C9	0.9954 (5)	−0.2408 (8)	0.8333 (5)	0.0551 (17)	
H91	1.0629	−0.2745	0.8340	0.062 (6)*	
C10	0.9358 (5)	−0.2017 (8)	0.7439 (5)	0.0462 (16)	
H101	0.9634	−0.2126	0.6844	0.062 (6)*	
C11	0.8401 (5)	−0.1490 (9)	0.7416 (4)	0.0485 (16)	
H111	0.8024	−0.1194	0.6807	0.062 (6)*	
C12	1.1069 (4)	−0.3021 (9)	1.0169 (5)	0.0521 (17)	
H121	1.1341	−0.3196	1.0856	0.062 (6)*	
H122	1.1398	−0.1911	0.9893	0.062 (6)*	
H123	1.1178	−0.4220	0.9803	0.062 (6)*	

### Atomic displacement parameters (Å^2^)

**Table:**

	*U*^11^	*U*^22^	*U*^33^	*U*^12^	*U*^13^	*U*^23^
S1	0.0519 (10)	0.0425 (8)	0.0543 (10)	−0.0001 (8)	0.0096 (8)	−0.0035 (8)
O1	0.066 (3)	0.044 (2)	0.052 (3)	0.000 (2)	0.008 (2)	−0.003 (2)
O2	0.042 (3)	0.055 (3)	0.055 (3)	0.011 (2)	0.011 (2)	0.005 (2)
O3	0.047 (3)	0.055 (2)	0.057 (3)	0.007 (2)	0.003 (2)	0.000 (2)
N1	0.045 (3)	0.037 (3)	0.045 (3)	0.002 (2)	0.007 (3)	0.002 (2)
N2	0.042 (3)	0.040 (3)	0.054 (3)	0.007 (2)	0.014 (3)	0.003 (3)
N3	0.056 (3)	0.039 (3)	0.045 (3)	0.002 (3)	0.008 (3)	0.000 (2)
N4	0.040 (3)	0.036 (3)	0.052 (3)	−0.004 (3)	0.008 (3)	−0.004 (2)
C1	0.042 (4)	0.039 (3)	0.054 (4)	−0.001 (3)	0.008 (3)	0.009 (3)
C2	0.053 (4)	0.035 (3)	0.040 (4)	0.002 (3)	0.019 (3)	−0.001 (3)
C3	0.050 (4)	0.040 (3)	0.046 (4)	0.002 (3)	0.010 (3)	0.004 (3)
C4	0.084 (5)	0.035 (4)	0.051 (4)	0.006 (3)	0.004 (4)	−0.006 (3)
C5	0.047 (4)	0.034 (3)	0.061 (5)	0.002 (3)	0.003 (4)	0.002 (3)
C6	0.045 (4)	0.038 (3)	0.061 (4)	−0.007 (3)	0.007 (4)	0.004 (3)
C7	0.057 (5)	0.043 (4)	0.053 (4)	−0.004 (3)	0.013 (4)	−0.002 (3)
C8	0.044 (4)	0.036 (3)	0.067 (5)	0.002 (3)	0.006 (4)	0.000 (3)
C9	0.059 (5)	0.045 (3)	0.064 (5)	−0.004 (3)	0.018 (4)	0.002 (3)
C10	0.047 (4)	0.043 (4)	0.054 (4)	−0.007 (3)	0.025 (4)	0.004 (3)
C11	0.051 (4)	0.047 (3)	0.047 (4)	−0.004 (3)	0.009 (3)	−0.005 (3)
C12	0.033 (4)	0.055 (4)	0.070 (4)	0.004 (3)	0.015 (3)	0.008 (3)

### Geometric parameters (Å, º)

**Table:**

S1—C1	1.694 (6)	C4—H42	0.9800
O1—C2	1.238 (6)	C4—H43	0.9800
O2—C7	1.383 (7)	C5—C6	1.427 (8)
O2—H2	0.8900	C5—H51	0.9500
O3—C8	1.372 (7)	C6—C11	1.427 (8)
O3—C12	1.409 (7)	C6—C7	1.428 (8)
N1—C1	1.391 (7)	C7—C8	1.393 (8)
N1—C2	1.401 (7)	C8—C9	1.380 (9)
N1—N2	1.422 (6)	C9—C10	1.413 (8)
N2—C5	1.275 (7)	C9—H91	0.9500
N3—C3	1.303 (7)	C10—C11	1.354 (8)
N3—N4	1.381 (6)	C10—H101	0.9500
N4—C1	1.295 (7)	C11—H111	0.9500
N4—H1	0.8800	C12—H121	0.9800
C2—C3	1.447 (8)	C12—H122	0.9800
C3—C4	1.503 (8)	C12—H123	0.9800
C4—H41	0.9800		
			
C7—O2—H2	107.6	N2—C5—H51	118.6
C8—O3—C12	117.9 (5)	C6—C5—H51	118.6
C1—N1—C2	122.5 (5)	C11—C6—C7	116.7 (6)
C1—N1—N2	117.3 (5)	C11—C6—C5	120.6 (6)
C2—N1—N2	120.1 (4)	C7—C6—C5	122.6 (6)
C5—N2—N1	115.2 (5)	O2—C7—C8	117.6 (6)
C3—N3—N4	114.9 (5)	O2—C7—C6	121.3 (6)
C1—N4—N3	128.8 (5)	C8—C7—C6	121.1 (6)
C1—N4—H1	115.6	O3—C8—C9	124.5 (6)
N3—N4—H1	115.6	O3—C8—C7	115.2 (6)
N4—C1—N1	115.2 (5)	C9—C8—C7	120.3 (6)
N4—C1—S1	123.2 (5)	C8—C9—C10	119.4 (6)
N1—C1—S1	121.6 (5)	C8—C9—H91	120.3
O1—C2—N1	120.3 (5)	C10—C9—H91	120.3
O1—C2—C3	125.5 (6)	C11—C10—C9	121.2 (6)
N1—C2—C3	114.2 (5)	C11—C10—H101	119.4
N3—C3—C2	124.1 (5)	C9—C10—H101	119.4
N3—C3—C4	116.6 (5)	C10—C11—C6	121.3 (6)
C2—C3—C4	119.2 (5)	C10—C11—H111	119.4
C3—C4—H41	109.5	C6—C11—H111	119.4
C3—C4—H42	109.5	O3—C12—H121	109.5
H41—C4—H42	109.5	O3—C12—H122	109.5
C3—C4—H43	109.5	H121—C12—H122	109.5
H41—C4—H43	109.5	O3—C12—H123	109.5
H42—C4—H43	109.5	H121—C12—H123	109.5
N2—C5—C6	122.8 (6)	H122—C12—H123	109.5

### Hydrogen-bond geometry (Å, º)

**Table:**

*D*—H···*A*	*D*—H	H···*A*	*D*···*A*	*D*—H···*A*
N4—H1···S1^i^	0.88	2.45	3.287 (5)	160
O2—H2···N2	0.89	1.87	2.662 (7)	146
O2—H2···N3^ii^	0.89	2.57	3.135 (7)	122

Symmetry codes: (i) −*x*+1, −*y*+1, −*z*+2; (ii) −*x*+1, −*y*, −*z*+2.
